# Impact of Carnivory on Human Development and Evolution Revealed by a New Unifying Model of Weaning in Mammals

**DOI:** 10.1371/journal.pone.0032452

**Published:** 2012-04-18

**Authors:** Elia Psouni, Axel Janke, Martin Garwicz

**Affiliations:** 1 Department of Psychology, Lund University, Lund, Sweden; 2 Center for Psychology, Kristianstad University, Kristianstad, Sweden; 3 LOEWE – Biodiversity and Climate Research Center – BiK-F, Senckenberganlage, Frankfurt am Main, Germany; 4 Goethe University, Institute for Ecology, Evolution & Diversity, Siesmayerstrasse, Frankfurt am Main, Germany; 5 Neuronano Research Center, BMC F10, Lund University, Lund, Sweden; University of Delaware, United States of America

## Abstract

Our large brain, long life span and high fertility are key elements of human evolutionary success and are often thought to have evolved in interplay with tool use, carnivory and hunting. However, the specific impact of carnivory on human evolution, life history and development remains controversial. Here we show in quantitative terms that dietary profile is a key factor influencing time to weaning across a wide taxonomic range of mammals, including humans. In a model encompassing a total of 67 species and genera from 12 mammalian orders, adult brain mass and two dichotomous variables reflecting species differences regarding limb biomechanics and dietary profile, accounted for 75.5%, 10.3% and 3.4% of variance in time to weaning, respectively, together capturing 89.2% of total variance. Crucially, carnivory predicted the time point of early weaning in humans with remarkable precision, yielding a prediction error of less than 5% with a sample of forty-six human natural fertility societies as reference. Hence, carnivory appears to provide both a necessary and sufficient explanation as to why humans wean so much earlier than the great apes. While early weaning is regarded as essentially differentiating the genus *Homo* from the great apes, its timing seems to be determined by the same limited set of factors in humans as in mammals in general, despite some 90 million years of evolution. Our analysis emphasizes the high degree of similarity of relative time scales in mammalian development and life history across 67 genera from 12 mammalian orders and shows that the impact of carnivory on time to weaning in humans is quantifiable, and critical. Since early weaning yields shorter interbirth intervals and higher rates of reproduction, with profound effects on population dynamics, our findings highlight the emergence of carnivory as a process fundamentally determining human evolution.

## Introduction

The evolutionary, ecological, social, behavioral and cognitive implications of the relatively high level of carnivory in humans compared to other extant primates [1] have been the subject of vigorous debates in a variety of research fields over the past fifty years [2], [3], [4], [5], [6], [7], [8], [9], [10]. In an evolutionary context, a ‘significant’ amount of carnivory has been suggested to correspond to a shift from 10% to 20% of food from meat [4]. In extant primate species, this shift corresponds to the difference between chimpanzees, with on average around 5% of their diet being meat [1], and tropical populations of hunter-gatherers living in environments similar to those of the African Pliocene, with estimated carnivorous diet of between 20% and 50% [4].

A crucial obstacle to reaching a consensus regarding the impact of carnivory on human development, life history and evolution is that its effects have been difficult to evaluate in quantitative terms [4], [5]. A case in point is the relatively short duration of lactation and suckling in humans in relation to other milestones in our life history [8], [11], [12], [13], [14] and as compared to the great apes [5], [15]. To date, factors that may have determined the timing of weaning in humans are poorly understood, resulting in a wide scatter of attempted predictions of ‘natural’ weaning age in humans from other life history variables [8], [11], [12], [13], [14]. However, as emphasized by syntheses of large numbers of studies, most of these predictions suggest a substantially later weaning age than practiced by modern humans, not only in the industrial world [16], but also in human natural fertility societies (the latter displaying an average of ca. 27 months [5]).

The early human weaning has implications not only for offspring development [5], but also for interbirth intervals [5], [17], [18] and thereby for the reproductive rate of the female, which in turn influences population dynamics and fitness of the species [19]. According to a longstanding hypothesis, the human weaning pattern was derived specifically from an ancestral hominid pattern [15] and is due to the introduction of meat into the diet of early hominins some 2.6-2.0 million years ago [4]. However, this hypothesis has not been possible to test since no model has been available for making a quantitative prediction of the consequences for time to weaning if a large brained primate species were to increase its intake of meat [4] (Text S1).

Relative time scales of early development appear to be very similar across mammals [20], [21], [22], [23] and lactation is a defining feature of Mammalia, common to all species of this class [24]. We therefore suggest that a potential key to understanding the timing of human weaning is to interpret it in a broad phylogenetic context by using a comparative analysis that includes not only hominids and other primates, but also species and traits representing other mammalian orders. The importance of a broad comparative perspective was emphasized by a recent radical reappraisal of another fundamental milestone in early human development – the timing of walking onset [23]. Our approach is quantitative and focused on the ontogenetic level of analysis [25], [26], in search for proximate causes for the timing of weaning (Text S2).

**Figure 1:**
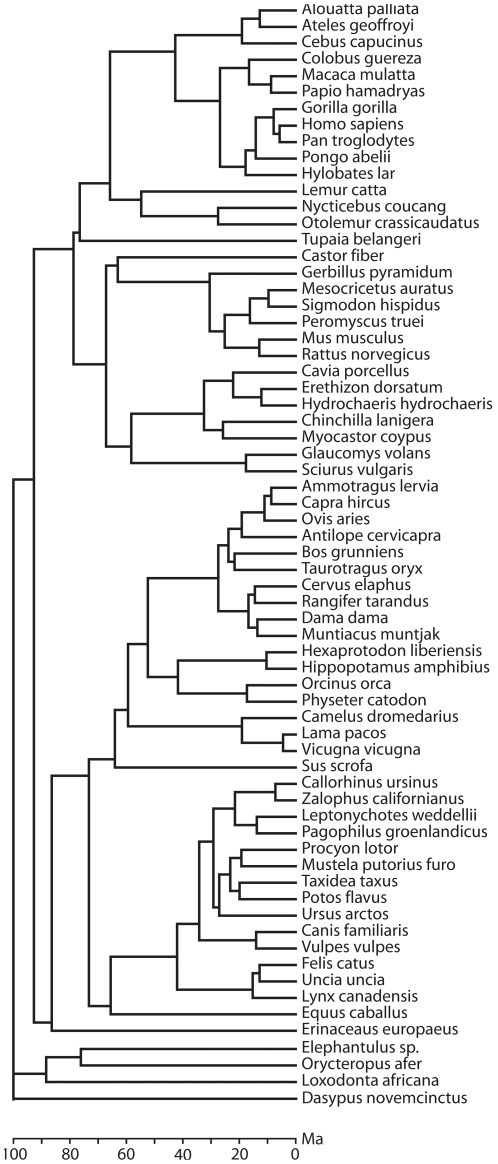
Phylogenetic relatedness and evolutionary divergence times of species in the present sample. Chronogram based on phylogenetic analysis complemented with phylogenomic data of the species in the present sample (N = 67) (Tables S1, S2, S3; see also Materials and Methods). The branch points indicate when in phylogeny different species diverged from each other and a time scale in Ma (Million years ago) is shown at the bottom.

In accordance with principles previously emphasized in the literature [11], [27], we developed a parsimonious, straight forward and biologically readily interpretable model. The model was based on sixty-seven species representing a wide taxonomic range of mammals and collected from twelve different orders (Fig. 1; Table S1). To avoid sample bias by overrepresentation of single lineages no more than one species was included from any given genus. Thus the 67 species in the sample represent 67 genera. A phylogenetic analysis and an independent contrasts analysis were performed to investigate if evolutionary dependence between the 67 species influenced the statistical analyses. The sample was carefully balanced for various species characteristics as outlined in Materials and Methods (Fig. S1). In line with previous literature, we employed adult brain mass [11], [28], [29], [30] and adult female body mass [11], [31], [32] as fundamental continuous independent variables that could potentially serve as predictors of time to weaning. Adult brain mass reflects the time during which the brain has developed during ontogenesis since mammalian brains develop at similar rates [33]. Therefore, if interspecies variation in weaning depends on brain mass, the duration of suckling may be assumed to reflect primarily the developmental time course and the needs of the offspring. If, on the other hand, interspecies variation in weaning depends on adult female body mass, it would primarily reflect the metabolic limitations of the lactating female [31]. The dependent variable was expressed either as time to weaning postnatal or post conception. Although the former measure is more conventional [5], [11], [31], [34], the latter appears biologically more relevant as it represents both the total developmental time of the offspring and the total time invested by the female.

Next, we categorized all species in our sample with respect to differences in limb biomechanics, dividing them into two groups – those that can assume a plantigrade standing position of the hindlimb and those that cannot (Table S1). Although it is currently not known exactly how limb biomechanics may influence the time course of motor development (Text S3), this dichotomous variable nevertheless accounts for a statistically significant amount of variance in the timing of walking onset, causing a grade shift [35] in the data set. In specific, species in the ‘plantigrade’ category systematically start walking later than those in the ‘non-plantigrade’ category [23]. Since walking onset is a fundamental developmental milestone, we wanted to explore in this study whether differences in limb biomechanics may also be associated with a systematic shift of other developmental events – such as weaning – along the ontogenetic time axis [23]. As an anatomical feature, the plantigrade standing position has a wide phylogenetic distribution and encompasses in the present sample all fourteen Primates and all fourteen Rodentia, as well as five of fourteen Carnivora and the five single species representing Macroscelidea, Scandentia, Erinaceomorpha, Cingulata and Tubulidentata (Fig. 1, Table S1). All other species, including digitigrade, unguligrade and those that have either rudimentary or lack external hindlimbs, were here categorized as ‘non-plantigrade’.

Finally, to allow a direct evaluation of the importance of dietary profile on the timing of weaning, we distinguished between carnivorous, omnivorous and herbivorous species [1], [24], defining ‘significant’ carnivory among primate species according to Foley's shift from 10% to 20% of food from meat [4], as described above. All data were obtained from the literature [5], [11], [24], [28], [36], [37], [38]. For clarity, the steps of the exploratory analysis underlying the final model are illustrated below.

## Results

Data on time to weaning, female body mass, adult brain mass, limb biomechanics and dietary profile from 67 species, representing 67 genera and 12 mammalian orders were included in the analyses. The N-numbers for the different categories with respect to limb biomechanics and dietary profile are presented in the relevant figures. For the majority of the species, the phylogenetic (Fig. 1) and independent contrast (Fig. S3) analyses were based on complete mitochondrial genome data (3680 amino acid sites).

**Figure 2:**
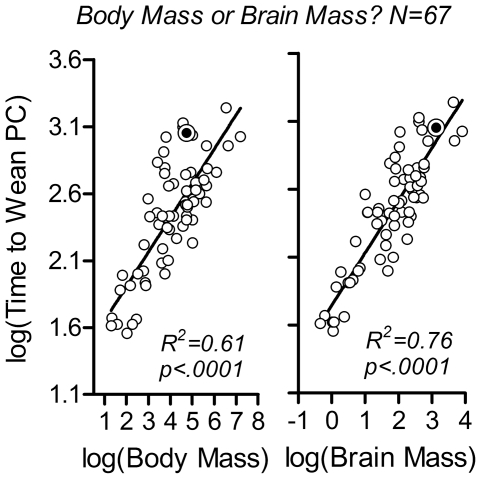
Brain mass is a better fundamental predictor of time to weaning than is female body mass. The continuous independent variables ‘female body mass’ and ‘adult brain mass’ were evaluated as potential predictors of time to weaning. Time to weaning (in days post conception; PC) was plotted as a function of female body mass (Body Mass, in grams) and adult brain mass (Brain Mass, in grams), in left and right panels, respectively. Note the log-log scales. Sample is as in Fig. 1 and Table S1 (N = 67). Double circle: humans (this value represents the mean value for 46 natural fertility societies; see Fig. 5 [5]). Solid line: Model II linear regression (reduced major axis) on all species. R^2^- and p-values are given in the diagrams. R^2^-values indicate amount of variance accounted for by the respective model. Adult brain mass accounted for a larger amount of variance in time to weaning, demonstrating that this parameter is a better predictor of time to weaning than is female body mass.

First, the two possible continuous independent variables were compared with respect to their relationship to the timing of weaning. Brain mass accounted for a substantially larger amount of variance in time to weaning than did body mass (Fig. 2) and the amount of variance accounted for was substantially larger when time to weaning was measured post conception rather than postnatal (Fig. S2). The partial correlation between brain mass and time to weaning was highly significant even when body mass was controlled for (original r_(67)_ = .87, p<.0001, r_partial_ = .69, p<.0001) and the influence of phylogenetic relatedness between the species on the correlation between brain mass and time to weaning post conception was minor (Materials and Methods; Fig. S3; Tables S1, S2). Since the contribution of phylogeny to the variance in the data was low, raw data were used throughout the subsequent analysis below.

**Figure 3:**
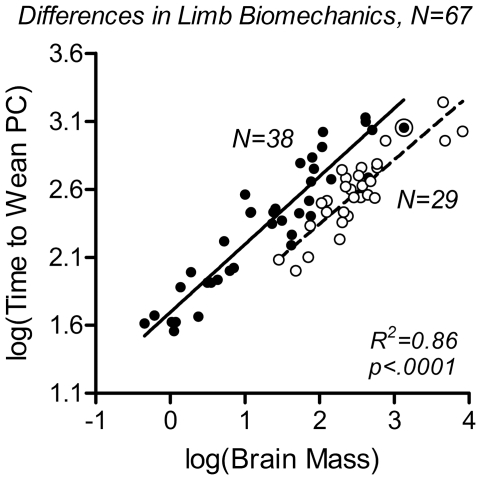
Limb biomechanics is a predictor of time to weaning. Time to weaning was plotted as a function of the continuous variable adult brain mass, log (Brain Mass), and the grouping variable ‘limb biomechanics’ (see main text and Materials and methods for definition; Table S1). Filled symbols and solid regression line represent species that can assume a plantigrade standing of the hindlimb (N = 38); open symbols and dashed regression line represent ‘non-plantigrade’ species, in the present sample including digitigrade and unguligrade species and those that have either rudimentary or completely lack external hindlimbs (N = 29). Double circle: humans. The grade shift between the two groups was highly significant. R^2^- and p-values for the multiple regression model are given in the diagram. The difference between this R^2^-value and the R^2^-value in Fig. 2 corresponds to the additional amount of variance accounted for by the grouping variable ‘limb biomechanics’.

**Figure 4:**
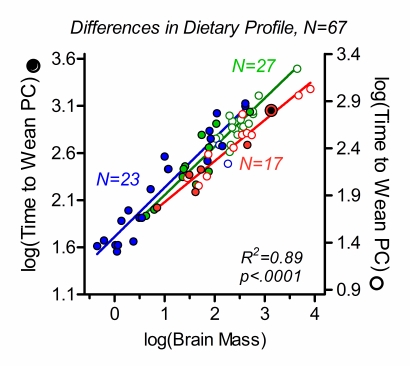
Dietary profile is a predictor of time to weaning. To illustrate the importance of the grouping variable ‘dietary profile’ independently of the grouping variable ‘limb biomechanics’, the grade shift shown in Fig. 3, of about 0.25 units along the Y-axis, has been compensated for by a shift between the left (filled circles; species that can assume plantigrade hindlimb position) and right (open circles; ‘non-plantigrade’ species, which cannot assume plantigrade hindlimb position) Y-axes. In this way, original data rather than values corrected for residual variance can be shown for both groups. Carnivorous, omnivorous and herbivorous species are shown red (N = 17), blue (N = 23) and green (N = 27), respectively. Double circle: humans. Solid lines: Model II linear regression (reduced major axis) on all species within in each dietary category (independently of limb biomechanics) are shown in matching colors. R^2^- and p-values from the multiple regression analysis described in main text are given in the diagrams. For full model equation see main text. The difference between this R^2^-value and the R^2^-value in Fig. 3 corresponds to the additional amount of variance accounted for by the grouping variable ‘dietary profile’.

Second, time to weaning as a function of brain mass was compared between the two categories of species with regard to limb biomechanics – species that can assume a plantigrade standing position of the hindlimb (lower extremity in humans), that is, stand on the full length of their hind foot including tarsal and metatarsal bones, and species that cannot. This categorization, which is associated with a systematic difference in the relative timing of walking onset [23], revealed a distinct grade shift in time to weaning, by a value of approximately 0.25 along the Y-axis (Fig. 3). Third, with the purpose to illustrate differences in time to weaning between species with a carnivorous, omnivorous or herbivorous diet, independently of limb biomechanics, species in the two categories of limb biomechanics were plotted on separate Y-axes and the two axes were shifted in relation to each other by a value of 0.25 (Fig. 4), thus preserving the original data. Analysis showed that carnivores systematically wean earlier than omnivores and herbivores. As omnivores and herbivores did not differ with regard to weaning time, they were subsequently pooled in a ‘non-carnivore’ group.

**Figure 5:**
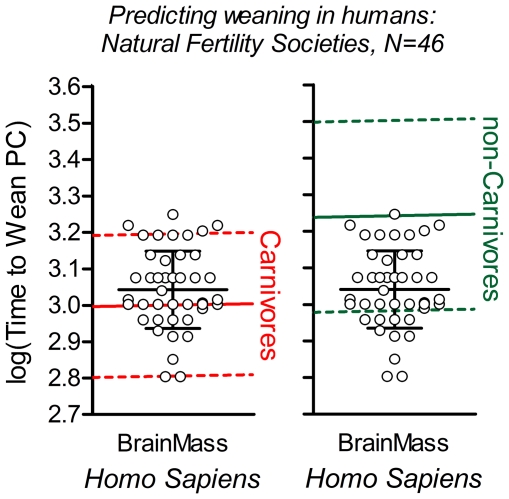
Time to weaning in humans is quantitatively predictable from a carnivorous diet. Predictions of time to weaning in humans based exclusively on species with a carnivorous (left panel, red) or a non-carnivorous diets (right panel, blue-green) were compared to a global sample of human natural fertility societies, shown as open circles (N = 46). Note that human Brain Mass is equal for all individual data points, but a scattered plot was used in order for the reader to be able to distinguish different data points with identical time to weaning. Solid colored lines: regression lines illustrating predicted mean values for time to weaning in carnivorous (red) and non-carnivorous (blue-green) species (omnivores and herbivores were pooled; cf. Fig. 4); dashed lines: 90% prediction lines. Long black horizontal line: mean value of time to weaning of the 46 human natural fertility societies; short black lines: +/−1SD [5]. See main text for prediction of time to weaning in humans based on the full model.

Fourth, the time to weaning predicted for a generic carnivore and non-carnivore with a brain mass equal to that of humans was compared to the actual time to weaning in a global sample of 46 human natural fertility societies [5] (Fig. 5). The sample fit the prediction based on the species in the carnivore group with regard to both mean value and distribution (left panel), but did not fit the prediction based on non-carnivores (right panel), thereby lending support to the hypothesis that carnivory may be a fundamental determinant of the early human weaning.

The final mixed factorial model – based on analysis of variance with covariance, ANCOVA – confirmed the highly significant main effects of ‘brain mass’ as continuous variable (F_(1, 66)_ = 461.29, p<.0001, partial η^2^ = .88), ‘limb biomechanics’ (F_(1, 66)_ = 42.27, p<.0001, partial η^2^ = .41) and ‘dietary profile’ (F_(1, 66)_ = 20.41, p<.0001, partial η^2^ = .25) as grouping variables, on ‘time to weaning’. The three independent variables together accounted for 89% of variance in ‘time to weaning’ (adjR^2^ = 0.89). The model equation was:
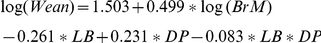
where ‘Wean’ is time to weaning in days post conception, ‘BrM’ is brain mass in grams, ‘LB’ = 1 for species that cannot assume a plantigrade standing position, and ‘DP’ = 1 for non-carnivorous species. Stepwise regression analysis determined the independent contributions of the three independent variables to 75.5%, 10.3% and 3.4% of variance explained respectively, in a highly significant model (F_(3, 66)_ = 177.12, p<.0001). Remarkably, the prediction for time to weaning in humans yielded by the model when based on all other sixty-six species (leaving humans out), was 1162 days post conception (ca 2 years and 5 months after birth). This is to be compared to the mean value of the forty six-human natural fertility societies illustrated in Fig. 5, which is 1129 days post conception (ca 2 years and 4 months after birth; with a range of 12 to 49 months). The prediction error of time to weaning in humans was thus less than 5%.

## Discussion

The model developed here involves twelve orders of mammals and allows for the first time a quantitative assessment of the possible effects of dietary profile on time to weaning in humans and sixty-six other species. As demonstrated by analysis of independent contrasts, the phylogenetic relatedness between the species in our sample had only minor effects on the significance of the results. Our findings indicate that dietary profile has had a profound evolutionary effect on weaning in mammals and that, if carnivory is taken into consideration, time to weaning is quantitatively predictable with remarkable precision in humans, despite our unique developmental features such as ‘secondary altriciality’ [35].

The remarkable precision of this prediction suggests that carnivory *per se* may provide not only a necessary but also a sufficient explanation for the difference between humans and the great apes with respect to the timing of weaning. Factors influencing diet quality, such as cooking [39], or behavioural and social factors influencing food abundance, such as alloparental or allomaternal help [40], may certainly have played important roles for aspects of human development and evolution in general or for human lactation practice and weaning patterns in particular [8]. However, in view of the high degree of similarity in relative time to weaning between humans and species that eat unprocessed meat and do not usually have helpers in parenting (Felidae, Mustelidae, Procyonidae, Ursidae; [40], as shown in the present analysis (cf. Table S1), it appears that neither human-specific food processing practices nor various forms of cooperative breeding have had a major influence on lactation duration *per se* in humans as a species. On the other hand, the importance of the different potential sources of variance in time to weaning, as outlined above, may be reflected in the large range of lactation durations across human societies and cultures [5], [16].

The impact of carnivory on time to weaning in humans and mammals in general demonstrated by our model supports the hypothesis that meat-eating even at levels below fully specialized carnivory may have had a major evolutionary effect on mammalian development and life history [4]. With respect to time to weaning specifically, our findings appear to confirm, on two accounts, the notion of a threshold effect of carnivory, postulated to correspond to a dietary shift from 10% to 20% of food from meat [1], [4]. First, we found no difference between herbivores and omnivores. Second, despite the moderate meat consumption of *Homo sapiens* [1], humans fit the prediction of time to weaning based on fully specialized carnivores although humans differ from these species with respect to gut anatomy, milk composition and suckling behaviour and are more similar to the great apes in these respects [2].

To relate to an ongoing debate in the field, brain mass, and by association brain development [33], was a better predictor of time to weaning than was body mass, suggesting that the timing of weaning reflects the developmental needs of the offspring rather than the metabolic limitations of the female. This is in contrast to previous studies of life history variation in primates, which have either indicated that brain mass and body mass serve equally well as predictors of time to weaning [11] or emphasized the importance of body mass [31] (Text S4; Fig. S4). On the other hand, our findings are compatible with the notion that brain mass accounts for a large amount of variance in mammalian life history in general [28], [29], [30] and in walking onset in particular [23]. Since weaning is a developmental milestone that follows walking onset and has, just like walking onset, immediate consequences for offspring independence, it is not surprising that the timing of the two appears to be determined by the same fundamental factors (Fig. S5).

In addition, the increased physical distance between mother and offspring after walking onset could potentially affect physiological mechanisms that sustain lactation. This may hypothetically further contribute to why species with a plantigrade standing position of the hindlimb, associated with a later walking onset [23], systematically wean later compared to ‘non-plantigrade’ species. However, the functional meaning of the two categories of limb biomechanics and the importance of walking onset as such should be interpreted with caution in the present context. It should also be noted that, in an analysis of walking onset across a wide range of mammals [23], time to walking onset post conception corresponded to gestation time for twelve out of the twenty-four species in the data base, since these species start walking soon after birth. These twelve species encompassed a few ‘plantigrade’ species and the unguligrade species in the sample, which constitute a subset of the ‘non-plantigrade’ category. By contrast, for the other twelve of the twenty-four species in the data base, the time from birth to walking onset was relatively long compared to gestation time, therefore constituting a substantial proportion (mean 39%, range 26–57%) of the total time to walking onset post conception [23]. These twelve species encompassed most ‘plantigrade’ species, including humans, and a small number of digitigrade species, which constitute a subset of the ‘non-plantigrade’ category.

In conclusion, our findings emphasize the high degree of similarity of relative time scales in development and life history of a wide phylogenetic range of mammals. Time to weaning appears to be determined by a limited set of factors across mammals in general, despite some 90 million years of evolution, and humans are no exception. Our findings underscore, in line with previous suggestions [35], that broad comparative models of human development and life history may be preferable or even necessary when evaluating the significance of features displayed by only one or a few species. Our model indicates that carnivory has a specific and quantifiable impact on human development and life history and, crucially, explains why *Homo* weans so much earlier than the great apes. Such an effect would have been impossible to evaluate in a model or data synthesis restricted to hominids or primates, which is an important reason why the ‘natural’ age of weaning in humans suggested by our model differs from that suggested by previous accounts [16].

The critical link between time to weaning and dietary profile adds to the general notion that the evolution of the hominids - and that of *Homo* in particular - was associated with a change towards higher-quality diet. Specifically, it has been proposed that with a given metabolic rate a large brain could have evolved only if another metabolically expensive tissue, such as the gut, would be reduced in size. But to maintain an energy intake sustaining that metabolic rate despite a reduced gut size, food quality must have been improved [41], for example by increased meat consumption. Our model suggests that the contribution of carnivory in this evolutionary context was to shorten the duration of lactation and suckling despite the overall prolongation of development associated with increased adult brain mass [14]. The resulting decreased interbirth intervals and increased rates of reproduction must have affected population dynamics profoundly. Our findings highlight therefore the emergence of carnivory as a process fundamentally determining human life history and evolution.

## Materials and Methods

To ensure a wide taxonomic range and taxonomic independence between individual species, the sample was drawn from 37 families representing 12 mammalian orders (Table S1; [24]), encompassing one species from any given genus, in order to avoid a sample bias by overrepresentation of single lineages. Thus, the 67 species in the sample represent 67 genera. The effect of phylogenetic relatedness between species on the statistical significance of the findings was minor and is accounted for as detailed below. The information was to a large extent taken from three previously well-established databases. The first provided data on brain mass [28], the second provided data on female body mass [37] and the third provided data on gestation time and weaning [36]. In six cases where the original database contained more than one species per genus [28], mean values for the genus were calculated for the continuous variables used in the analysis (Table S1). Brain mass data for primates were obtained from a different database, focused exclusively on this order [11].

The sample was approximately balanced with respect to (a) the four orders representing large (>200) numbers of species: Rodentia, Carnivora, Primates, and Artiodactyla, in addition to a series of single representatives of orders with relatively few (<20) species, referred to as ‘Others’ below (Table S1, Fig. S1); (b) species that can (N = 38) and those that cannot (N = 29) assume a plantigrade stance with their hindlimb (lower extremity in humans), reflecting differences in limb biomechanics [24]. This distinction accounts for a statistically significant grade shift in the timing of walking onset [23] and may therefore also be associated with a systematic shift of other developmental milestones along the ontogenetic time axis; (c) carnivorous (N = 17), omnivorous (N = 23) and herbivorous (N = 27) species [24].

There was substantial variance in body mass and brain mass within orders and substantial overlap between orders (except Rodentia), with a particularly close match between Carnivora and Primates with respect to brain mass (Fig. S1). To match the brain mass of the largest herbivore (*Loxodonta africana*) and to widen the overall range of brain mass in the sample, two particularly large brained carnivorous species (of the Cetacea) were included [38]. When tested, their exclusion from the analysis was inconsequential for the overall results.

The phylogenetic relationships among the 67 species were reconstructed from 3680 amino acid (aa) sites of sequence data from 12 H-strand encoded protein coding genes of mitochondrial (mt) genome and the cytochrome *b* gene sequences for a few species (see Table S2 for details). The sequences were aligned and the program ProtTest version 1.3 [42] suggested mtMAM+4Γ+I model of sequence evolution [43], [44] for the ML analyses. Some branches were constrained in accordance with mitogenomic and phylogenomic analyses of 3 Mbp of sequence data [45], [46] for avoiding an erroneous tree topology due to reconstruction artefacts from short sequences. An un-rooted maximum likelihood (ML) tree and branch lengths and divergence times were reconstructed with the TreeFinder (TF) program package [47]. The tree was oriented according to phylogenomic analyses [45] for divergence time estimates and depicting the tree. The phylogenetic position of the beaver (*Castor fiber*), European Polecat (*Mustela putorius*), and the long-tailed chinchilla (*Chinchilla lanigera*) needed to be constrained, probably because these species were only represented by cytochrome *b* data (380 aa). Divergence time estimates were based on 11 calibration points (see Table S3), [48] and the log-NPRS method as implemented in TF [47].

The possible dependence of the findings from the brain mass and weaning-time parameters on evolutionary relatedness [49] was determined by an independent contrast analysis implemented in the Mesquite program package [50] and the PDAP:PDTREE module [51] using the topology, branch lengths, log brain mass and log weaning time post conception as parameters. The independent contrast analysis did not suggest dependency of the brain mass and weaning time characters with the animals' evolutionary history (Fig. S3).

## Supporting Information

Text S1 **Predicting the effect of carnivory on time to weaning.** A clarification of why it is not obvious that carnivory would yield earlier weaning rather than later.
(DOC)

Text S2 **The functional and the evolutionary biologists' perspective.** Drawing attention to two complementary views on causes for the timing of weaning: proximate and ultimate.
(DOC)

Text S3 **The plantigrade and the non-plantigrade limb.** Presenting a hypothesis how limb biomechanics may affect the developmental time to walking onset.
(DOC)

Text S4 **Measuring developmental time as postnatal vs. post conception in primates.** A brief account of why the importance of measuring developmental time from conception may be obscured when analysis is restricted to primates.
(DOC)

Figure S1 **Sample characteristics per mammalian order.** Scatter plots showing distributions of female body mass (Body Mass), left, and adult brain mass (Brain Mass), right, in the present sample (Table S1), broken down into different orders. ‘Others’ refers to a collection of individual species belonging to orders with relatively small numbers of species. Horizontal lines indicate mean values (long) and +/−1SD (short). For clarity, symbols alternate between orders.
(PDF)

Figure S2 **Counting time from birth.** The continuous independent variables female body mass, log (Body Mass), in left panel, and adult brain mass, log (Brain Mass), in right panel, as predictors of time to weaning, log (Time to Wean), when expressed in days postnatal (PN). g: grams. Sample as in Table S1; double circle: humans. Solid lines: Model II linear regression (reduced major axis) on all species (N = 67); R^2^- and p-values given in diagram should be compared to those in Fig. 2, which shows the corresponding data set, but with time to weaning expressed in days post conception.
(PDF)

Figure S3 **Effects of phylogenetic relatedness.** The influence of phylogenetic relatedness on the statistical significance of the findings illustrated in Fig. 2 (right panel) was evaluated. Number of contrasts: 66. Solid line: Model II linear regression (reduced major axis). The Pearson correlation coefficient was 0.79 (F_(1, 64)_ = 103.9, p<.0001), showing that the effects of phylogenetic relatedness were minor.
(PDF)

Figure S4 **Separate analysis of the primates in the present sample.** The continuous independent variables female body mass, log (Body Mass), in left panel, and adult brain mass, log (Brain Mass), in right panel, as predictors of time to weaning log (Time to Wean) in the primates of the present sample (N = 14, Table S1). Time to weaning expressed in days post conception (PC) in upper diagrams (compare to Fig. 2) and as days postnatal (PN) in lower diagrams (compare to Fig. S2). g: grams. Double circle: humans. Solid lines: Model II linear regression (reduced major axis) on all fourteen species; R^2^- and p-values given in diagrams.
(PDF)

Figure S5 **Ratio of time to walking/time to weaning.** Walking onset is determined mainly by adult brain mass [23]. If weaning were determined by adult female body mass, the ratio between walking onset (Walk) and time to weaning (Wean) would vary as a function of the ratio between adult brain mass (Brain Mass) and female body mass (Body Mass). This is, however, not the case. Humans display one of the highest values of Brain mass/Body mass ratio in a wide taxonomic range of ground walking mammals [23], but have a Walk/Wean ratio that is close to the mean for these mammals. Left panel: log (Walk/Wean) plotted as a function of log (Brain Mass/Body Mass), (N = 23). Double circle: humans. Solid line: Model II linear regression (reduced major axis) on all species; R^2^- and p-values given in diagram. Right panel: Aligned dot plot showing mean and +/−1SD for log (Walk/Wean) in the sample in left panel. Dotted line indicates the value for humans to facilitate comparison between diagrams.
(PDF)

Table S1 **Sample of species and data used for analysis.** BrM: Adult brain mass, grams; BoM: Adult female body mass, grams; Gest: Gestation time, days; Wean: Time to weaning, days postnatal; LB: Limb biomechanics; pl: can assume plantigrade standing position of the hindlimb; n-pl: non-plantigrade – cannot assume plantigrade standing position of the hindlimb, including digitigrade, unguligrade and species that have either rudimentary or lack external hindlimbs. DP: Dietary profile; C: Carnivore; O: Omnivore; H: Herbivore. Three species in the sample have delayed implantation during pregnancy. For these, as for all other species, the value in the column ‘Gest’ represents total time from conception to birth. Of the species that can assume a plantigrade standing position of the hindlimb, only few actually walk with plantigrade posture. Most ‘plantigrade’ species walk and run with digitigrade posture in which the heel does not contact, or apply force to, the substrate. Elephants are listed ‘non-plantigrade’ because their heel is supported above the ground by a large connective tissue pad. During walking force transmission through this pad makes elephants mechanically plantigrade.
(DOC)

Table S2 **Accession numbers and scientific names of species included in the analysis.** Order as in the tree in Fig. 1. mt: mitochondrial genome, cyt b: cytochrome oxidase b gene sequence.
(DOC)

Table S3 **Calibration points use for the construction of the tree in** **Fig. 1** **.** *The split denotes the branch where the species-pair shares a last common ancestor. **For the TF algorithm one calibration points needs to be fixed. The fixed date was taken from: Hallström BM and Janke A. (2010) Mammalian evolution may not be strictly bifurcating. Mol Biol Evol 27:2804–2816; see also Materials and Methods.
(DOC)
