# Supplementary material for: Impact of Carnivory on Human Development and Evolution Revealed by a New Unifying Model of Weaning in Mammals
Source: PLoS One. 2012 Apr 18;7(4):e32452. doi: 10.1371/journal.pone.0032452 (PMC3329511; doi:10.1371/journal.pone.0032452)
Supplement: Text S1 — Predicting the effect of carnivory on time to weaning. A clarification of why it is not obvious that carnivory would yield earlier weaning rather than later. (DOC) [file pone.0032452.s001.doc]

**Text S1**: It has often been assumed that carnivory would yield earlier weaning [6]. However, it has also been argued that meat-eating would instead result in later weaning due to the positive effects on the nutritious state of the lactating female, which would in turn increase the nutritious value of the maternal milk and make it appropriate also for older offspring [4]. Later weaning in humans as compared to the great apes would also be consistent with the general prolongation of other phases of life history [14] associated with the large brain of the human species [11], [29].
