# Supplementary material for: Impact of Carnivory on Human Development and Evolution Revealed by a New Unifying Model of Weaning in Mammals
Source: PLoS One. 2012 Apr 18;7(4):e32452. doi: 10.1371/journal.pone.0032452 (PMC3329511; doi:10.1371/journal.pone.0032452)
Supplement: Text S2 — The functional and the evolutionary biologists' perspective. Drawing attention to two complementary views on causes for the timing of weaning: proximate and ultimate. (DOC) [file pone.0032452.s002.doc]

**Text S2**: A functional biologist, focusing on the ontogenetic level of analysis, would regard for instance the pace of brain development as a (proximate) cause for the timing of weaning. An evolutionary biologist, on the other hand, might instead regard the pace of brain development as a product of selection, reflecting for instance evolutionary adaptations to juvenile mortality rates, which would then constitute the ultimate cause for the timing of weaning. It is important to emphasize that these two perspectives are complementary rather than contradictory [25], [26] and that focus of the present study was on the former and not the latter.
