# Supplementary material for: Impact of Carnivory on Human Development and Evolution Revealed by a New Unifying Model of Weaning in Mammals
Source: PLoS One. 2012 Apr 18;7(4):e32452. doi: 10.1371/journal.pone.0032452 (PMC3329511; doi:10.1371/journal.pone.0032452)
Supplement: Text S3 — The plantigrade and the non-plantigrade limb. Presenting a hypothesis how limb biomechanics may affect the developmental time to walking onset. (DOC) [file pone.0032452.s003.doc]

**Text S3:** There are general biomechanical differences between plantigrade and non-plantigrade limbs, regardless of whether or not a given species that can assume a plantigrade standing position actually walks or runs in a plantigrade fashion. These differences pertain to the relative length of the foot, but also to the mobility of the limb and, therefore, to its biomechanical degrees of freedom. It is possible that for adequate motor control, the smaller range of possible movements in limbs that cannot assume a plantigrade standing position requires learning or adaptation of fewer muscle synergies in the course of ontogenetic development. If that is the case, a given level of motor performance should hypothetically take relatively less time to achieve in species with such limbs, which would explain why species with plantigrade limbs display a relatively later walking onset [23].
