# Supplementary material for: Impact of Carnivory on Human Development and Evolution Revealed by a New Unifying Model of Weaning in Mammals
Source: PLoS One. 2012 Apr 18;7(4):e32452. doi: 10.1371/journal.pone.0032452 (PMC3329511; doi:10.1371/journal.pone.0032452)
Supplement: Text S4 — Measuring developmental time as postnatal vs. post conception in primates. A brief account of why the importance of measuring developmental time from conception may be obscured when analysis is restricted to primates. (DOC) [file pone.0032452.s004.doc]

**Text S4**: Similar to the twelve-order mammalian sample as a whole, brain mass accounted for a larger amount of variance in time to weaning than did body mass for the primates only in our sample (compare Fig. S4, upper panel and Fig. 2). By contrast, there was a striking difference between the twelve-order mammalian sample as a whole and primates only with regard to the amount of variance accounted for when time to weaning was measured as post conception compared to postnatal (compare Fig. S4, lower panel and Fig. S2). Measuring developmental time from conception has previously been shown to be a crucial step towards a unifying model of walking onset in a wide range of mammals, including humans [23]. The same approach appears to capture a general mammalian pattern also with regard to timing of weaning, but is obscured in a sample of primates only, presumably as a result of similarities in relative gestation time between relatively closely related species, which renders the amount of variance in time to weaning accounted for independent of whether time is measured from birth or conception.
