# Supplementary figures and images for: Impact of Carnivory on Human Development and Evolution Revealed by a New Unifying Model of Weaning in Mammals
Source: PLoS One. 2012 Apr 18;7(4):e32452. doi: 10.1371/journal.pone.0032452 (PMC3329511; doi:10.1371/journal.pone.0032452)

*Sample characteristics per mammalian order, N=67*

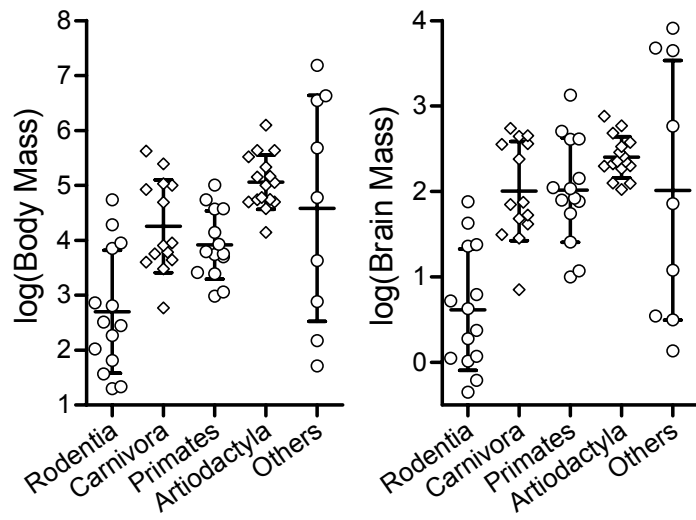

Supplement: Figure S1 — Sample characteristics per mammalian order. Scatter plots showing distributions of female body mass (Body Mass), left, and adult brain mass (Brain Mass), right, in the present sample (Table S1), broken down into different orders. ‘Others’ refers to a collection of individual species belonging to orders with relatively small numbers of species. Horizontal lines indicate mean values (long) and +/−1SD (short). For clarity, symbols alternate between orders. (PDF) [file pone.0032452.s005.pdf]

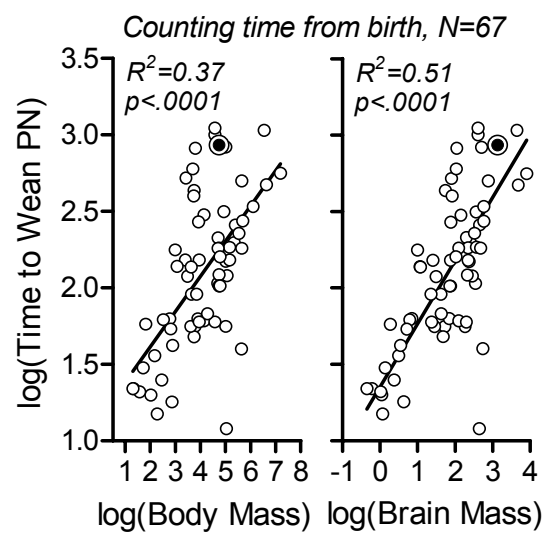

Supplement: Figure S2 — Counting time from birth. The continuous independent variables female body mass, log (Body Mass), in left panel, and adult brain mass, log (Brain Mass), in right panel, as predictors of time to weaning, log (Time to Wean), when expressed in days postnatal (PN). g: grams. Sample as in Table S1; double circle: humans. Solid lines: Model II linear regression (reduced major axis) on all species (N = 67); R2- and p-values given in diagram should be compared to those in Fig. 2, which shows the corresponding data set, but with time to weaning expressed in days post conception. (PDF) [file pone.0032452.s006.pdf]

*Effects of phylogenetic relatedness, N=66*

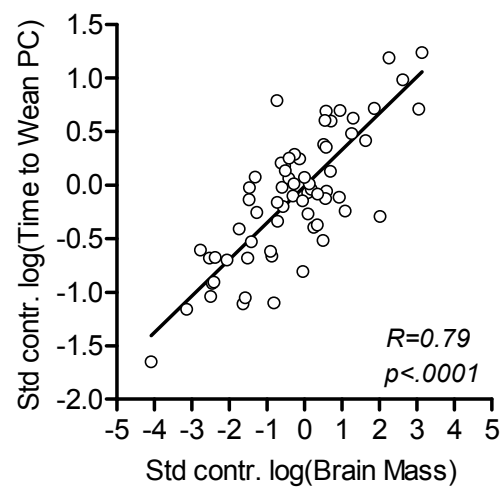

Supplement: Figure S3 — Effects of phylogenetic relatedness. The influence of phylogenetic relatedness on the statistical significance of the findings illustrated in Fig. 2 (right panel) was evaluated. Number of contrasts: 66. Solid line: Model II linear regression (reduced major axis). The Pearson correlation coefficient was 0.79 (F(1, 64) = 103.9, p<.0001), showing that the effects of phylogenetic relatedness were minor. (PDF) [file pone.0032452.s007.pdf]

Primates in present sample only, N=14

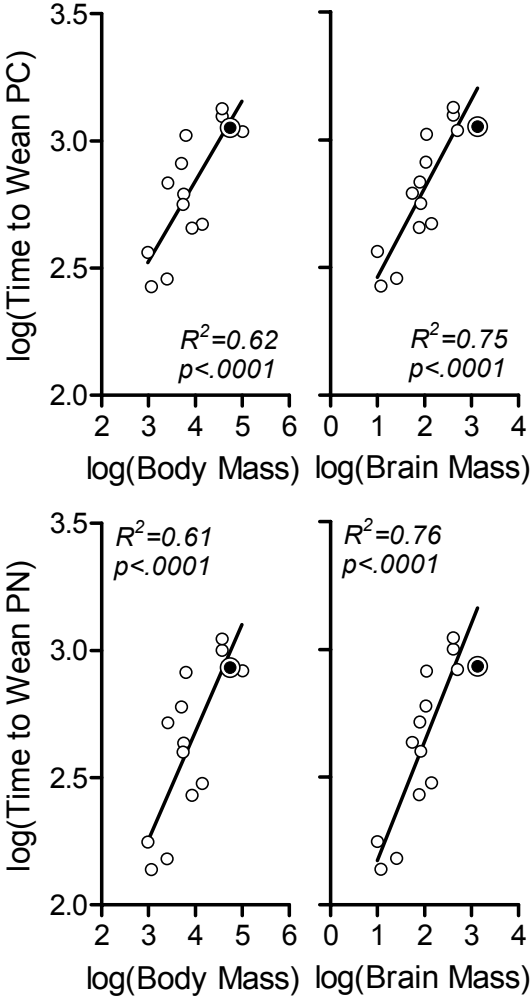

Supplement: Figure S4 — Separate analysis of the primates in the present sample. The continuous independent variables female body mass, log (Body Mass), in left panel, and adult brain mass, log (Brain Mass), in right panel, as predictors of time to weaning log (Time to Wean) in the primates of the present sample (N = 14, Table S1). Time to weaning expressed in days post conception (PC) in upper diagrams (compare to Fig. 2) and as days postnatal (PN) in lower diagrams (compare to Fig. S2). g: grams. Double circle: humans. Solid lines: Model II linear regression (reduced major axis) on all fourteen species; R2- and p-values given in diagrams. (PDF) [file pone.0032452.s008.pdf]

*Ratio time to walking:time to weaning, N=23*

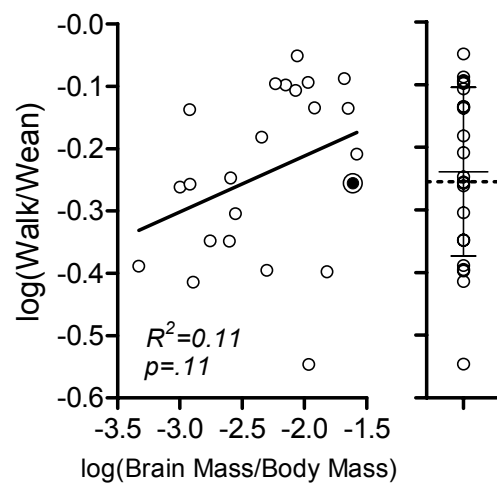

Supplement: Figure S5 — Ratio of time to walking/time to weaning. Walking onset is determined mainly by adult brain mass [23]. If weaning were determined by adult female body mass, the ratio between walking onset (Walk) and time to weaning (Wean) would vary as a function of the ratio between adult brain mass (Brain Mass) and female body mass (Body Mass). This is, however, not the case. Humans display one of the highest values of Brain mass/Body mass ratio in a wide taxonomic range of ground walking mammals [23], but have a Walk/Wean ratio that is close to the mean for these mammals. Left panel: log (Walk/Wean) plotted as a function of log (Brain Mass/Body Mass), (N = 23). Double circle: humans. Solid line: Model II linear regression (reduced major axis) on all species; R2- and p-values given in diagram. Right panel: Aligned dot plot showing mean and +/−1SD for log (Walk/Wean) in the sample in left panel. Dotted line indicates the value for humans to facilitate comparison between diagrams. (PDF) [file pone.0032452.s009.pdf]
