# Supplementary material for: Impact of Carnivory on Human Development and Evolution Revealed by a New Unifying Model of Weaning in Mammals
Source: PLoS One. 2012 Apr 18;7(4):e32452. doi: 10.1371/journal.pone.0032452 (PMC3329511; doi:10.1371/journal.pone.0032452)
Supplement: Table S1 — Sample of species and data used for analysis. BrM: Adult brain mass, grams; BoM: Adult female body mass, grams; Gest: Gestation time, days; Wean: Time to weaning, days postnatal; LB: Limb biomechanics; pl: can assume plantigrade standing position of the hindlimb; n-pl: non-plantigrade – cannot assume plantigrade standing position of the hindlimb, including digitigrade, unguligrade and species that have either rudimentary or lack external hindlimbs. DP: Dietary profile; C: Carnivore; O: Omnivore; H: Herbivore. Three species in the sample have delayed implantation during pregnancy. For these, as for all other species, the value in the column ‘Gest’ represents total time from conception to birth. Of the species that can assume a plantigrade standing position of the hindlimb, only few actually walk with plantigrade posture. Most ‘plantigrade’ species walk and run with digitigrade posture in which the heel does not contact, or apply force to, the substrate. Elephants are listed ‘non-plantigrade’ because their heel is supported above the ground by a large connective tissue pad. During walking force transmission through this pad makes elephants mechanically plantigrade. (DOC) [file pone.0032452.s010.doc]

**Table S1**

| **Order** | **Family** | **Genus/*species*** | **BrM^1^** | **BoM^2^** | **Gest^3^** | **Wean^3^** | **LB^4^** | **DP^4^** |
| --- | --- | --- | --- | --- | --- | --- | --- | --- |
| Carnivora | Felidae | *Panthera [mean]** | 220 | 113450 | 104 | 154,3 | n-pl | C |
| Carnivora | Felidae | *Lynx canadensis* | 75 | 8900 | 63 | 152 | n-pl | C |
| Carnivora | Mustelidae | *Taxidea taxus* | 53 | 6050 | 210 | 56 | pl | C |
| Carnivora | Procyonidae | *Potos flavus* | 31,2 | 3000 | 115 | 119 | pl | C |
| Carnivora | Procyonidae | *Procyon lotor* | 41,6 | 4400 | 63 | 91 | pl | C |
| Carnivora | Ursidae | *Ursus [mean]*** | 450 | 244933 | 230 | 258 | pl | C |
| Carnivora | Canidae | *Vulpes vulpes* | 48 | 5662 | 52 | 48 | n-pl | C |
| Carnivora | Felidae | *Felis catus* | 28,4 | 4000^1^ | 65 | 56 | n-pl | C |
| Carnivora | Canidae | *Canis lupus familiaris* | 70,2 | 8000^1^ | 63 | 63^4^ | n-pl | C |
| Carnivora | Mustelidae | *Mustela putorius (furo)* | 7,1^5^ | 589 | 42 | 63 | pl | C |
| Carnivora | Otariidae | *Callorhinus ursinus* | 355 | 49100 | 240 | 107 | n-pl | C |
| Carnivora | Otariidae | *Zalophus californianus* | 363 | 84666 | 259 | 315 | n-pl | C |
| Carnivora | Phocidae | *Pagophilus groenlandicus* | 442 | 107000^1^ | 350 | 12 | n-pl | C |
| Carnivora | Phocidae | *Leptonychotes weddellii* | 550 | 421666 | 304 | 40 | n-pl | C |
| Primates | Lemuridae | *Lemur catta* | 25,6^6^ | 2466 | 135 | 152 | pl | H |
| Primates | Loridae | *Nycticebus coucang* | 10^6^ | 966 | 188 | 177 | pl | O |
| Primates | Galagonidae | *Otolemur crassicaudatus* | 11,8^6^ | 1143 | 130 | 138 | pl | O |
| Primates | Cebidae | *Alouatta palliata* | 55,1^6^ | 5659 | 186 | 433 | pl | H |
| Primates | Cebidae | *Cebus capucinus* | 79,2^6^ | 2602 | 162 | 521 | pl | O |
| Primates | Cebidae | *Ateles geoffroyi* | 111^6^ | 6265 | 232 | 821 | pl | O |
| Primates | Cercopithecidae | *Macaca [mean]**** | 83,6^6^ | 5494 | 165 | 399,5 | pl | O |
| Primates | Cercopithecidae | *Papio hamadryas* | 143^6^ | 14028 | 171 | 300 | pl | O |
| Primates | Cercopithecidae | *Colobus polykomos* | 76,7^6^ | 8508 | 185 | 270 | pl | H |
| Primates | Hylobatidae | *Hylobates lar* | 108^6^ | 5050 | 217 | 600 | pl | H |
| Primates | Hominidae | *Pongo pygmaeus* | 413^6^ | 37115 | 249 | 1003 | pl | O |
| Primates | Hominidae | *Pan troglodytes* | 410^6^ | 37618 | 229 | 1111 | pl | O |
| Primates | Hominidae | *Gorilla gorilla gorilla* | 506^6^ | 101386 | 256 | 834 | pl | H |
| Primates | Hominidae | *Homo sapiens* | 1350 | 55000 | 270 | 859^7^ | pl | C^8^ |
| Artiodactyla | Suidae | *Sus scrofa* | 185 | 100900 | 115 | 56 | n-pl | O |
| Artiodactyla | Hippopotamidae | *Hippopotamus amphibius* | 590 | 1258333 | 234 | 341 | n-pl | H |
| Artiodactyla | Hippopotamidae | *Hexaprotodon liberiensis* | 260 | 215000 | 199 | 198 | n-pl | H |
| Artiodactyla | Camelidae | *Lama glama* | 225 | 142500 | 330 | 152 | n-pl | H |
| Artiodactyla | Camelidae | *Vicugna vicugna* | 198 | 50000 | 340 | 213 | n-pl | H |
| Artiodactyla | Cervidae | *Muntiacus muntjak* | 124 | 14000 | 210 | 61 | n-pl | O |
| Artiodactyla | Cervidae | *Dama dama* | 223 | 54500 | 233 | 183 | n-pl | H |
| Artiodactyla | Cervidae | *Cervus [mean]***** | 374 | 145666,5 | 239 | 184,5 | n-pl | H |
| Artiodactyla | Cervidae | *Rangifer tarandus* | 288 | 113200 | 228 | 120 | n-pl | H |
| Artiodactyla | Bovidae | *Taurotragus oryx* | 480 | 432500 | 274 | 182 | n-pl | H |
| Artiodactyla | Bovidae | *Bos grunniens* | 334 | 333000 | 274 | 228 | n-pl | H |
| Artiodactyla | Bovidae | *Antilope cervicapra* | 200 | 37500 | 168 | 60 | n-pl | H |
| Artiodactyla | Bovidae | *Ammotragus lervia* | 210 | 55500 | 149 | 122 | n-pl | H |
| Artiodactyla | Bovidae | *Ovis aries* | 125 | 50000 | 146 | 182 | n-pl | H |
| Artiodactyla | Bovidae | *Capra hircus* | 106 | 60000 | 155 | 160 | n-pl | H |
| Artiodactyla | Camelidae | *Camelus dromedarius* | 762^5^ | 434000 | 405 | 500 | n-pl | H |
| Rodentia | Sciuridae | *Sciurus vulgaris* | 6,23 | 324 | 38 | 62 | pl | H |
| Rodentia | Sciuridae | *Glaucomys volans* | 1,89 | 65 | 40 | 58 | pl | O |
| Rodentia | Castoridae | *Castor [mean]****** | 42,5 | 19303 | 117 | 68 | pl | H |
| Rodentia | Muridae | *Peromyscus [mean]******* | 0,62 | 21,5 | 25 | 22 | pl | O |
| Rodentia | Cricetidae | *Mesocricetus auratus* | 1,12 | 105 | 16 | 20 | pl | O |
| Rodentia | Muridae | *Gerbillus pyramidum* | 1,04 | 37 | 21 | 21 | pl | O |
| Rodentia | Muridae | *Rattus norvegicus* | 2,38 | 280 | 21 | 25 | pl | O |
| Rodentia | Muridae | *Mus musculus* | 0,45 | 20 | 19 | 22 | pl | O |
| Rodentia | Muridae | *Sigmodon hispidus* | 1,18 | 185 | 27 | 15 | pl | O |
| Rodentia | Erethizontidae | *Erethizon dorsatum* | 24 | 9000 | 210 | 60 | pl | H |
| Rodentia | Caviidae | *Cavia porcellus* | 4,28 | 728 | 68 | 18 | pl | H |
| Rodentia | Hydrochaeridae | *Hydrochaeris hydrochaeris* | 76 | 55000 | 150 | 103 | pl | H |
| Rodentia | Chinchillidae | *Chinchilla lanigera* | 5,25 | 642 | 111 | 54 | pl | O |
| Rodentia | Echimyidae | *Myocastor coypus* | 23 | 7150 | 131 | 91 | pl | H |
| Perissodactyla | Equidae | *Equus caballus* | 585^5^ | 484000^1^ | 337 | 274 | n-pl | H |
| Proboscidea | Elephantidae | *Loxodonta africana* | 4480 | 3507000 | 670 | 1071 | n-pl | H |
| Macroscelidea | Macroscelididae | *Elephantulus myurus* | 1,37 | 52 | 46^1^ | 30^2^ | pl | O |
| Scandentia | Tupaiidae | *Tupaia belangeri* | 3,15 | 150^1^ | 46 | 36 | pl | O |
| Erinaceomorpha | Erinaceidae | *Erinaceus europaeus* | 3,5 | 771 | 40 | 42 | pl | O |
| Cingulata | Dasypodidae | *Dasypus novemcinctus* | 12 | 4300 | 133 | 137 | pl | O |
| Tubulidentata | Orycteropodidae | *Orycteropus afer* | 72^5^ | 60000 | 225 | 103 | pl | O |
| Cetacea | Delphinidae | *Orcinus orca* | 4780^9^ | 4300000 | 435 | 471 | n-pl | C |
| Cetacea | Physeteridae | *Physeter catodon* | 8183^9^ | 15400000 | 502 | 560 | n-pl | C |
|  |  |  |  |  |  |  |  |  |
| Means in table above: | | **P tigris* | 302 | 119700 | 105 | 121 |  |  |
|  |  | **P leo* | 260 | 139500 | 109 | 216 |  |  |
|  |  | **P pardus* | 157 | 42325 | 97 | 110 |  |  |
|  |  | ***U maritimus* | 500 | 286366 | 230 | 303 |  |  |
|  |  | ***U arctos* | 400 | 203500 | 225 | 213 |  |  |
|  |  | ****M mulatta* | 95,1 | 5413 | 165 | 292 |  |  |
|  |  | ****M maura* | 72 | 5575 | 165 | 507 |  |  |
|  |  | *****C elaphus* | 365 | 120333 | 245 | 156 |  |  |
|  |  | *****C unicolor* | 383 | 171000 | 232 | 213 |  |  |
|  |  | ******C canadensis* | 40 | 19606 | 128 | 60 |  |  |
|  |  | ******C fiber* | 45 | 19000 | 105 | 76 |  |  |
|  |  | *******P leucopus* | 0,63 | 23 | 26 | 22 |  |  |
|  |  | *******P maniculatus* | 0,6 | 20 | 24 | 22 |  |  |
|  |  |  |  |  |  |  |  |  |

**References, as indexed:**

1. Sacher GA, Staffeldt EF (1974) Relation of Gestation Time to Brain Weight for Placental Mammals: Implications for the Theory of Vertebrate Growth. Am Nat 108:593-615.

2. Ernest SKM (2003) Life History Characteristics of Placental Nonvolant Mammals: Ecological Archives E084-093. Ecology 84:3402.

3. de Magalhaes JP, Costa J (2009) A database of vertebrate longevity records and their relation to other life-history traits. J Evol Biol 22:1770-1774.

4. Grzimek's Animal Life Encyclopedia M. Hutchins, Evans A.V., Jackson J.A., Kleiman, D.G., Murphy, J.B., Thoney, D.A., et al, Ed. (Gale, Detroit, 2003), vol. 13-16.; cf. University of Michigan Museum of Zoology, Animal Diversity Web http://animaldiversity.ummz.umich.edu/site/index.html

5. Garwicz M, Christensson M, Psouni E (2009) A unifying model for timing of walking onset in humans and other mammals. Proc Natl Acad Sci U S A 106:21889-21893.

6. Harvey PH, Clutton-Brock TH (1985) Life History Variation in Primates. Evolution 39:559-581.

7. Kennedy GE (2005) From the ape's dilemma to the weanling's dilemma: early weaning and its evolutionary context. J Hum Evol. 48:123-145.

8. Stanford CB (2001) A Comparison of Social Meat-Foraging by Chimpanzees and Human Foragers. In: Stanford CB, Bunn HT, editors. Meat-Eating & Human Evolution. Oxford: Oxford University Press. pp 122-140

9. Lefebvre L, Marino L, Sol D, Lemieux-Lefebvre S, Arshad S (2006) Large brains and lengthened life history periods in Odontocetes. Brain Behav Evol 68:218-228.
