# Supplementary material for: Impact of Carnivory on Human Development and Evolution Revealed by a New Unifying Model of Weaning in Mammals
Source: PLoS One. 2012 Apr 18;7(4):e32452. doi: 10.1371/journal.pone.0032452 (PMC3329511; doi:10.1371/journal.pone.0032452)
Supplement: Table S2 — Accession numbers and scientific names of species included in the analysis. Order as in the tree in Fig. 1. mt: mitochondrial genome, cyt b: cytochrome oxidase b gene sequence. (DOC) [file pone.0032452.s011.doc]

**Table S2**

| **Data** | **Acc. Nr.** | **Species** |
| --- | --- | --- |
| cytb | AY065883 | *Alouatta palliata* |
| cytb | AY065903 | *Ateles geoffroyi* |
| mt | AJ309866 | *Cebus albifrons (capucinus)* |
| mt | AY863427 | *Colobus guereza* |
| mt | AJ309865 | *Macaca sylvanus* |
| mt | Y18001 | *Papio hamadryas* |
| mt | X93347 | *Gorilla gorilla* |
| mt | X93334 | *Homo sapiens* |
| mt | X93335 | *Pan troglodytes* |
| mt | X97707 | *Pongo abelii* |
| mt | X99256 | *Hylobates lar* |
| mt | AJ309867 | *Nycticebus coucang* |
| mt | AJ421453 | *Tupaia belangeri* |
| mt | AB371093 | *Otolemur crassicaudatus* |
| mt | AJ421451 | *Lemur catta* |
| cytb | DQ088706 | *Castor fiber* |
| cytb | AJ851269 | *Gerbillus gerbillus* |
| mt | EU660218 | *Mesocricetus auratus* |
| cytb | AF425227 | *Sigmodon hispidus* |
| cytb | AF108703 | *Peromyscus truei* |
| mt | J01420 | *Mus musculus* |
| mt | AJ428514 | *Rattus norvergicus* |
| cytb | GU136732 | *Cavia porcellus* |
| cytb | FJ357428 | *Erethizon dorsatum* |
| cytb | GU136721 | *Hydrochoerus hydrochaeris* |
| cytb | AF464760 | *Chinchilla lanigera* |
| cytb | EU544663 | *Myocastor coypus* |
| cytb | AF157921 | *Glaucomys volans* |
| mt | AJ238588 | *Sciurus vulgaris* |
| mt | EF466060 | *Ammotragus lervia* |
| mt | GU229280 | *Capra hircus* |
| mt | AF010406 | *Ovis aries* |
| mt | AP003422 | *Antilope cervicapra* |
| mt | EF494179 | *Bos grunniens* |
| cytb | AF036278 | *Tragelaphus oryx* |
| mt | AB245427 | *Cervus elaphus* |
| mt | AB245426 | *Rangifer tarandus* |
| cytb | AJ000022 | *Dama dama* |
| mt | AY225986 | *Muntiacus muntjak* |
| cytb | Y08814 | *Hexaprotodon liberiensis* |
| mt | AJ010957 | *Hippopotamus amphibius* |
| mt | GU187202 | *Orcinus orca* |
| mt | AJ277029 | *Physeter macrocephalus* |
| mt | EU159113 | *Camelus dromedarius* |
| mt | AJ566364 | *Lama pacos* |
| mt | FJ456892 | *Vicugna vicugna* |
| mt | AJ002189 | *Sus scrofa* |
| mt | AM181016 | *Callorhinus ursinus* |
| mt | AM181017 | *Zalophus californianus* |
| mt | AM181025 | *Leptonychotes weddellii* |
| mt | AM181030 | *Pagophilus groenlandicus* |
| mt | AM711899 | *Procyon lotor* |
| cytb | AB026107 | *Mustela putorius* |
| cytb | AF057132 | *Taxidea taxus* |
| mt | AF303110 | *Ursus arctos* |
| mt | EU789780 | *Canis lupus familiaris* |
| mt | GQ374180 | *Vulpes vulpes* |
| mt | DQ660304 | *Potos flavus* |
| mt | U20753 | *Felis catus* |
| mt | EF551004 | *Uncia uncia* |
| mt | AH014070 | *Lynx canadensis* |
| mt | X79547 | *Equus caballus* |
| mt | X88898 | *Erinaceus europaeus* |
| mt | AB096867 | *Elephantulus sp.* |
| mt | Y18475 | *Orycteropus afer* |
| mt | DQ316069 | *Loxodonta africana* |
| mt | Y11832 | *Dasypus novemcinctus* |
| mt | X83427 | *Ornithorhynchus anatinus* |
| mt | Z29573 | *Didelphis virginiana* |
| mt | Y10524 | *Macropus robustus* |
