# Supplementary material for: Impact of Carnivory on Human Development and Evolution Revealed by a New Unifying Model of Weaning in Mammals
Source: PLoS One. 2012 Apr 18;7(4):e32452. doi: 10.1371/journal.pone.0032452 (PMC3329511; doi:10.1371/journal.pone.0032452)
Supplement: Table S3 — Calibration points use for the construction of the tree in Fig. 1 . *The split denotes the branch where the species-pair shares a last common ancestor. **For the TF algorithm one calibration points needs to be fixed. The fixed date was taken from: Hallström BM and Janke A. (2010) Mammalian evolution may not be strictly bifurcating. Mol Biol Evol 27:2804–2816; see also Materials and Methods. (DOC) [file pone.0032452.s012.doc]

**Table S3**

| Split* | Max Ma | Min Ma |
| --- | --- | --- |
| Homo sapiens – Loxodonta africana | 100 (fixed**) | - |
| Homo sapiens – Equus caballus | 131.5 | 61.5 |
| Homo sapiens – Lemur catta | 65.8 | 55.6 |
| Homo sapiens – Macaca sylvanus | 34 | 23.5 |
| Homo sapiens – Pan troglodytes | 10 | 5.7 |
| Felis catus – Canis lupus familiaris | 65.8 | 39.7 |
| Equus caballus – Canis lupus familiaris | 131.5 | 62.5 |
| Equus caballus – Erinaceus europaeus | 131.5 | 62.5 |
| Physeter macrocephalus – Bos grunniens | 65.8 | 52.4 |
| Cavia porcellus – Sciurus vulgaris | 65.8 | 55.6 |
| Mus musculus – Rattus norvergicus | 14 | 10.4 |
